# Receptive to an authoritative voice? Experimental evidence on how patronizing language and stressing institutional sources affect public receptivity to nutrition information

**DOI:** 10.1016/j.ssmph.2022.101295

**Published:** 2022-11-19

**Authors:** Tim van Meurs, Joost Oude Groeniger, Willem de Koster, Jeroen van der Waal

**Affiliations:** a Department of Public Administration and Sociology, Erasmus University Rotterdam, Burgemeester Oudlaan 50, 3062PA, Rotterdam, the Netherlands; b Department of Public Health, Erasmus MC, Doctor Molewaterplein 40, 3015GD, Rotterdam, the Netherlands

**Keywords:** Health communication, Health inequalities, Institutional information, Psychological reactance, Receptivity, Source derogation

## Abstract

Common strategies to make official nutrition information more persuasive include highlighting its institutional sources and using simple and direct language. However, such strategies may be counterproductive, as institutions are no longer self-evidently deemed to be legitimate in contemporary societies and such language can be viewed as patronizing. Our preregistered, population-based survey experiment fielded among a high-quality Dutch probability sample in February 2022 (*n* = 1947) 1) examines whether these dominant strategies hold up when tested against suggestions of psychological reactance and source derogation, and 2) scrutinizes if such responses are stronger among less-educated citizens. Our experiment mirrored real-life examples of health-information campaigns concerning healthy and unhealthy beverages, with data collected on seven outcome measures to discern receptivity toward the information and its sources. We found that just highlighting institutional sources in the information did not lead to it being perceived more negatively. This was also the case when the language used could be perceived as patronizing, with reactance only present for one outcome measure. Moreover, while less-educated citizens were generally less receptive to nutrition information (six of seven outcome measures), versions that could possibly be perceived as patronizing or/and highlighted institutional sources did not make them less receptive systematically. Importantly, therefore, while our results show that the dominant health-communication strategies do not increase receptivity either, their use will probably not have a negative effect on the general public and so do not need to be discarded.

## Highlights

• Preregistered randomized population-based survey experiment.
• Studies effects of communication strategies on receptivity to nutrition information.
• Receptivity is not negatively affected by dominant communication strategies.
• No systematic educational differences in the effects of the strategies.

## 1 Introduction

Many societies across the globe deal with problems arising from overweight or obesity. In 2016, about forty percent of adults globally was overweight (WHO, 2021), and more recent statistics show that this goes for more than half the EU population (Eurostat, 2021). In their attempts to tackle this, public-health organizations have produced numerous initiatives to encourage citizens to improve their diet, including providing information on nutrition (see e.g., Brambila-Macias et al., 2011; Rimal & Lapinski, 2009; Snyder, 2007). Such information, broadly, is meant to communicate to citizens what healthy and less healthy food choices are, in order to motivate or empower them to make conscious decisions about their food behavior (Schiavo, 2007). Health-communication scholars have long studied how this can be done effectively (e.g., Noar, 2006), yet there are indications that some of the dominant strategies may be counter-productive (e.g., Fransen et al., 2015; Rains, 2013).

Common advice in relation to the provision of health communication is to do so through authoritative sources or present it as information from expert sources. (Cummings, 2014; Gehrau et al., 2021). As such, many members of the European Public Health Nutrition Alliance (EPHNA)^1^ – the network of official bodies providing nutrition communication organizations in Europe – highlight the scientific background of their information prominently on their various webpages. Furthermore, to emphasize the official nature of the advice, it is often disseminated by governmental organizations and linked to specific health professionals or bodies (e.g., De Dobbelaer et al., 2018), since this is assumed to improve a message's credibility (Cummings, 2014).

Nevertheless, in highly individualized societies, institutions no longer have self-evident legitimacy. Instead, their actions are more critically reflected on by (Beck & Beck-Gernsheim, 2002; Houtman et al., 2011, 2021). As such, citizens’ adherence to institutions is more strongly based on an interplay of various individual and contextual characteristics, instead of the more taken-for-granted adherence of generations before. That makes explicitly signaling the involvement of institutional sources not as unquestionably beneficial as theorized. Indeed, a critical attitude toward institutions may, in fact, be one of the reasons for the low uptake of official health information, as previously highlighted for receptivity to, e.g., information about nutrition (Van Meurs et al., 2022b), the hazards of smoking (Veldheer et al., 2019) or COVID-19 (Caplanova et al., 2021; Wong & Jensen, 2020), or public support for various non-health related institutional interventions (e.g., Davidovic & Harring, 2020; Rudolph, 2009).

Attempts to further improve the uptake of health information can often be seen in the use of simplified language (e.g., Calderón & Beltran, 2005; Meppelink et al., 2015), and explicitness of the message (Dillard & Shen, 2005; Miller et al., 2007); making directly clear what the intention of the message is in an understandable way. However, the way this is done could lead to health messages coming across as patronizing (Atkinson & Sloan, 2017), portraying a sense of perceived superiority while actually trying to be helpful. This can take the form of sentences like *‘Everyone should know this*: there is too much sugar in sugar-sweetened beverages’, or ‘*It would be smart* to eat more fruit’. As patronizing language in health information has been found to be unattractive (Brown & Draper, 2003), the strategy of making it easier to understand and more direct may unintentionally lower the public's receptivity to it. Indeed, earlier studies exploring receptivity to health information have suggested that various health-communication strategies may actually increase the extent to which the advice being provided is rejected (e.g., Dillard & Shen, 2005; for an overview, see Rains, 2013). However, since many of these studies only involved small non-probability samples, it is unclear what the relevance of the aversion identified is to how health information is received among the public at large.

To advance the research described above, we conducted a survey experiment among a high-quality panel randomly drawn from the official Dutch population register. The goal was to test the public's receptivity to two communication strategies: 1) the highlighting of institutional sources, and 2) the use of simplified and direct language that could be perceived as patronizing. Our focus was on a specific form of nutrition information – the intake of healthy drinks. The consumption of sugar-sweetened beverages (SSBs) is a major cause of excess weight (Thompson et al., 2009), and the issue is therefore highlighted in various health-information campaigns. As knowledge of the risks of SSB has been shown to be associated with their consumption (Park et al., 2014), information that disseminates the facts may have a positive effect on the levels of sugar consumed.

A correlation between the consumption of excess sugar and an individual's educational attainment has also been identified (Thompson et al., 2009), with less-educated citizens found to be more likely to (over)consume SSBs than their more-educated counterparts. Ideally, therefore, health-promotion efforts should have an impact on this group to an at least equal, but preferably greater, extent than on more-educated citizens. However, research has shown that less-educated individuals are less receptive to nutrition information than more-educated individuals (Van Meurs et al., 2022c), with this gap strongly associated with the former's more negative views toward institutions (Van Meurs et al., 2022b). This may be an indication that the two strategies tested in the present study will be less well received by this group.

This study examines how the aforementioned communication strategies affect citizens' receptivity to nutrition information among the general public. In addition, we test for heterogeneous treatment effects by educational attainment (Mullinix et al., 2015). As such, we aim to answer a two-part question: *Does highlighting institutional sources and the use of patronizing language in nutrition information affect the public's receptivity to it, and does this differ between less- and more-educated individuals?* The study is positioned in the Netherlands, where information provision is a widely-employed health promotion strategy and an official organization is used to disseminate such guidance (Voedingscentrum; Netherlands Nutrition Centre^2^). The provision of health information also has a prominent role within the National Prevention Agreement, a highly ambitious comprehensive collaboration between the Dutch government and over 70 civil society organizations. Its goal is to improve the health of Dutch citizens, including by reducing the number of those who are overweight and obese by more than ten percent over the next two decades (Rijksoverheid, 2018).

## 2 Theory

### 2.1 Common health-communication elements: institutional sources and patronizing language

Nutrition information is a form of persuasive communication aiming to encourage people to eat and drink more healthily. In many countries, an official organization is responsible for disseminating such guidance population-wide. An example is the EPHNA, of which 17 members communicate advice on nutrition on a national or regional level. These organizations collaborate with, or are part of, national health, governmental, and science institutions, which is emphasized when they produce their advice on nutrition. Some focus on the fact that their guidance is backed by science, e.g., the Flemish Institute for Health Living assures readers that its ‘food triangle’ is “scientifically proven” (Gezond Leven, n.d.) and the Spanish Academy of Nutrition and Dietetics stresses its use of an in-house scientific committee. Other members have a direct link to national governmental agencies: the Austrian Agency for Health and Food Safety and the German Federal Centre for Nutrition are both part of federal offices, and the Netherlands Nutrition Centre is an independent organization funded by two ministries.

These institutional connections are emphasized to increase the persuasiveness and credibility of the information provided (Cummings, 2014; Gehrau et al., 2021). Cummings (2014) argues that although citizens can understand health advice, they cannot always judge which information is ‘right’: “Rather than communicating advice about health risks in a manner that is divorced from the expert base that generated this advice, public health officials *should seek wherever possible to reveal this base* so that it may be rationally evaluated by the public” (p. 1054, emphasis added). In other words, an institutional background should be communicated to signal expertise and officiality.

Nonetheless, the success of this strategy likely hinges on the extent to which the institutions are regarded as legitimate. The longstanding process of individualization has, however, led to many of these institutions losing some of their self-evident legitimacy (Beck & Beck-Gernsheim, 2002; Houtman et al., 2011, 2021); it “can no longer be simply taken for granted or expected; it has to be worked on and won” (Meyer et al., 2008, p.179). This was clearly visible during the COVID-19 pandemic, where an initial upsurge of trust in both government and science (Oude Groeniger et al., 2021) was quickly followed by a more critical stance (Bromme et al., 2022). Clearly, then, at a time when the legitimacy of institutions cannot be taken for granted, highlighting explicit connections to them may not be as effective as anticipated.

Keeping information simple and direct is another common strategy (Calderón & Beltran, 2005), e.g., using brief, easy to understand, sentences and placing an exaggerated emphasis on the key points. This probably has merit, since it is often argued that health information is less effective among those in the lower social strata for reasons such as cognitive factors (Van Meurs, Çoban, De Koster, Van der Waal, & Oude Groeniger, 2022a, Van Meurs, Oude Groeniger, De Koster, & Van der Waal, 2022c). Nevertheless, this type of language is sometimes also criticized for seeming to talk down to people who do not follow the guidelines being communicated (Atkinson & Sloan, 2017).

While the focus of current research into potentially patronizing health advice is on communication with elderly citizens (e.g., Atkinson & Sloan, 2017), health information aimed at the general public includes many of the same elements. In guidelines on nutrition, some material signals that unhealthier diets are the ‘wrong’ or ‘irrational’ choice by claiming: “*you don't need* products such as candy, snacks and soft drinks *at all* for your health” (Voedingscentrum, n.d. emphasis added); “*We all actually already know* our shelves are full of unhealthy food” (Alliantie Voeding voor de Gezonde Generatie, n.d. emphasis added); and “The message is *reasonably simple*: if we are gaining weight, we need to eat less and be more active!” (European Food Information Council, 2017). These examples seem to imply that people who do not follow the guidance are somehow ‘wrong’ or ‘not smart enough’, potentially causing them to feel stigmatized.

If persuasive communication is to be effective at changing behavior, it is important that the information is viewed positively. Communication strategies like those described above are often employed in an attempt to achieve this, but may also cause the content being conveyed to be perceived negatively. The foremost evidence of this comes from studies on psychological reactance and source derogation.

### 2.2 Opposing the message: reactance

Individuals confronted with persuasive communication might show signs of reactance: a motivational state in which people feel the need to (actively) reject a message as a way to regain the freedom they perceive to be under threat (Brehm & Brehm, 1981). Reactance goes beyond just passively ignoring the message, instead representing a state in which the communicated content produces a negative reaction and, possibly, outcomes that are polar opposite to those hoped for and expected.

Information interventions are often lauded for their low level of intrusiveness (Diepeveen et al., 2013), i.e., individuals still have agency to choose how to act upon the guidance being given. Nevertheless, as reactance theory shows, the intention to change behavior into the ‘proper’ conduct conveyed in health advice may cause recipients to in fact feel as if their freedom to choose is threatened (Brehm & Brehm, 1981). Accordingly, people may feel pressure to adopt a particular form of behavior when it is not reflective of how they actually behave in real life. Moreover, the stronger the perceived intention to correct, the stronger this sense of threat is likely to be (Dillard & Shen, 2005).

In view of the issues highlighted above, attempts to increase the persuasiveness of a message may be counterproductive. Indeed, early accounts of reactance have already reported that seemingly more-credible sources lead to more negative receptivity (Brehm, 1966), although this is not a consistent finding (e.g., Rains & Turner, 2007). Nevertheless, given the critical attitudes toward institutions that are common in today's individualized societies (e.g., Houtman et al., 2021), stressing the involvement of such sources may be perceived as unwelcome correction, rather than as a sign of greater credibility. Consequently, people might oppose, rather than comply with, the information directed at them.

Similarly, health-information language that is perceived to be patronizing may be unwelcome (Brown & Draper, 2003) and likely heightens message recipients’ perceptions that their current behavior – if not in line with that communicated– is inappropriate. Again, the sense of being judged that arises from the use of patronizing language also probably breeds an aversion to, rather than compliance with, the information being conveyed.

According to reactance theory, this opposition can be expressed in various ways. Recipients may perceive the information as a greater threat to their freedom to choose if it stresses institutional sources and uses patronizing language. They may then become defiant and expressively negative about the advice – defined as state reactance – and the communicated ‘appropriate’ behavior. As a worst-case effect, such information may subdue the envisioned impact, or even influence the recipients in the opposite way to that intended (Oschatz et al., 2021; Zhao & Fink, 2021). This leads to our first four hypotheses:

*The perceived threat to freedom is greater after reading information that a) stresses its institutional sources, or b) stresses its institutional sources and uses patronizing language, than it is after reading basic information* (hypothesis 1).

*State reactance is greater after reading information that a) stresses its institutional sources, or b) stresses its institutional sources and uses patronizing language, than it is after reading basic information* (hypothesis 2).

*Attitudes to reducing the consumption of sugar-sweetened beverages are more negative after reading information that a) stresses its institutional sources, or b) stresses its institutional sources and uses patronizing language, than it is after reading basic information* (hypothesis 3).

*Intended non-compliance is greater after reading information that a) stresses its institutional sources, or b) stresses its institutional sources and uses patronizing language, than it is after reading basic information* (hypothesis 4).

### 2.3 Beyond the message: source derogation

Aside from aversion to the message in question, how information is presented may also influence opinions of its sources, and even of information provision in general (Fransen et al., 2015). One possible negative effect is source derogation, i.e., rejecting the validity of the institution as a source of information (Cameron et al., 2002). Institutions’ absence of self-evident legitimacy in contemporary societies may also have a negative impact on attitudes to the sources themselves and their information in general, creating a feedback loop. Indeed, research has shown that compliance is greater when institutions are considered legitimate (Tyler & Jackson, 2014). This suggests that a more strongly negative evaluation of institutions (i.e. source derogation) may cause further undermining of their broader health-promotion efforts in the future.

How sources of information are evaluated commonly comes down to identification and appreciation. Identification focuses on whether those providing the advice resemble the intended audience in relevant ways (Chang, 2011; McCroskey et al., 1975). Indeed, it is often the case that a source is judged to be more credible when it is also perceived to be similar to the recipient (Hu & Sundar, 2010; Wright, 2000). Consequently, interventions are more effective when they communicate the experiences of like-minded individuals (Van Meurs et al., 2022a). This is in contrast to institutionalized and patronizing information. Institutional sources are, by definition, impersonal and, as a result, unlike many recipients; and patronizing language causes further distance, giving those the advice is intended for the impression that they are being talked down to. Consequently, we expect that:

*Source disidentification is higher after reading information that a) stresses its institutional sources, or b) stresses its institutional sources and uses patronizing language, than it is after reading basic information* (hypothesis 5).

The appreciation of sources is often based on their competence, trustworthiness and benevolence (McCroskey & Teven, 1999). Extant research has shown that information from explicitly named sources is not deemed to be more credible than that where these sources are not identified (Bates et al., 2006). Moreover, given the critical attitudes to institutions today, this strategy may increase the negativity of perceptions. This is particularly the case when the language used is condescending (König & Jucks, 2019) and thus links this type of communication to the named institutional sources, causing them to be judged more negatively. Accordingly, we hypothesize:

*Source disappreciation is higher after reading information that a) stresses its institutional sources, or b) stresses its institutional sources and uses patronizing language, than it is after reading basic information* (hypothesis 6).

Lastly, as a culmination of the theorizing above, we hypothesize that institutional and patronizing information may negatively affect attitudes toward nutrition information provision in general. In line with negative receptivity to the information and source, it may also take the form of overall disdain for any advice that aims to persuade recipients to change their diet. Our final main effect hypothesis is thus:

*Attitude to information provision as an effort to promote health is more negative after reading information that a) stresses its institutional sources, or b) stresses its institutional sources and uses patronizing language, than it is after reading basic information* (hypothesis 7).

### 2.4 Educational differences in reactance and source derogation

Less-educated individuals make less use of health information than their more-educated counterparts (Koç & van Kippersluis, 2017). Moreover, when it is taken into account, it is generally not as effective among the former group (Van Meurs et al., 2022c). This gap is strongly associated with the anti-institutionalism of less-educated individuals (Van Meurs et al., 2022b), meaning that the use of named institutional sources is potentially counterproductive. Moreover, given the well-documented high levels of distrust of institutions felt by this group, whether toward government (Noordzij et al., 2021a), science (Achterberg et al., 2017) or healthcare institutions (Laveist et al., 2009), a strategy of overtly communicating the involvement of such sources may result in less receptivity to a message by less-educated individuals in particular.

Alongside this, the lifestyles of less-educated individuals are often frowned on by their more-educated individuals (Bourdieu, 1984; Currid-Halkett, 2017), leading less-educated individuals to perceive that their lifestyles are being stigmatized (Kuppens et al., 2018) and that they are not represented by institutions populated by the latter (Lamont, 2018; Noordzij et al., 2021b). Any attempts to interfere in their lives may thus be perceived as patronizing. Indeed, it is argued that initiatives by a dominant group that seek to ‘edify’ the dominated have an aura of power dynamics, with the former being accused of believing that they have the moral superiority to decide what is best for the latter (Jackman, 1994; Veldheer et al., 2019). These power dynamics are also very much in play in health inequalities (Bergman et al., 2020; McCartney et al., 2020) and have a role in conflicts between educational groups (Stubager, 2009). When the language in information that already condemns certain lifestyle elements is also patronizing, it probably increases the perception that the behavior is regarded as morally wrong, reflecting the stigmatization that less-educated individuals already feel.

Given the above, we expect the information effects theorized in hypothesis 1 to 4 to be moderated by educational attainment:

*The effects hypothesized in H1-4 are stronger among less-educated individuals than among their more-educated counterparts* (hypothesis 8i-a/8i-b – hypothesis 8iv-a/8iv-b).

We expect similar outcomes for source effects. First, institutions like those central in health information are largely composed of more-educated individuals (Rivera, 2012). Lifelong socialization in elite institutions (especially higher education) allows those in this group to identify more closely with them (Forster & van de Werfhorst, 2020; Lareau, 2015), whereas less-educated individuals lack this experience and are thus likely to identify with them less.

Second, less-educated individuals judge the more educated to be less benevolent, and no more competent that their own in-group (Spruyt & Kuppens, 2015). Consequently, this group's more negative assessments of information providers are likely due to: a perceived closer connection between (the lifestyles of) the more-educated and the institutional sources mentioned in the health advice; or the use of condescending language in that advice, which is experienced as stigmatizing health behavior that is typically more associated with less-educated individuals. We thus theorize in relation to the source effects in hypothesis 5 and 6 that:

*The effects hypothesized in H5-6 are stronger among less-educated individuals than among their more-educated counterparts* (hypothesis 8v-a/8v-b – hypothesis 8vi-a/8vi-b).

Lastly, it is likely that the effects of institutional and patronizing advice on attitudes toward the provision of nutrition information in general are experienced more negatively by less-educated citizens, who probably take it to be just a continuation of the stigmatization they feel is a constant presence in their daily lives. Information that feeds this stigmatization may thus strengthen the view that the provision of nutrition information is another way to look down on them and their lifestyles. Accordingly, and reflecting the expected effect moderation theorized in hypothesis 7, our final hypothesis is as follows:

*The effects hypothesized in H7 are stronger among less-educated individuals than among their more-educated counterparts* (hypothesis *8vii*-a/8vii-b).

## 3 Data and methods

This study was preregistered with the Open Science Framework (OSF) and received ethical approval from our institution's ethics review board (blinded for peer review) before data collection. The details of the preregistration can be found at: https://osf.io/we82u?view_only=d1b16852011d4a5fa7730f09048983c6 3

### 3.1 Study participants

The participants were recruited from the LISS (Longitudinal Internet Studies for the Social Sciences) panel, which is administered by Centerdata (Tilburg University, the Netherlands). The panel is composed of a true probability sample of households taken from the official Dutch population register and comprises about 7500 individuals. Households are approached via recruitment letters, phone calls, or home visits, and to reduce non-response, households are contacted up to 15 times, and an extensive refusal conversion process is applied to maximize cooperation. Respondents are paid for their participation and computers and internet connections are provided to those who need them to ensure the panel is representative. Centerdata also recruits refreshment samples, sometimes oversampling hard-to-reach groups, to improve representation. An individual response rate of 80.4% was reached in 2019 (Centerdata, 2020).

In the current study, fielded in February 2022, Dutch adults (aged 18 and above) were sampled from the panel, with a response rate of 80.9%. Of these individuals (*n* = 2340), we only selected those who spent more than 10 seconds on the web page with the experimental condition, as this was determined to be the minimum amount of time required to read the text. This produced a final sample of 2092 respondents.

### 3.2 Study design

We used a survey experiment with a between-subjects design. The respondents were asked to complete an online survey and, once they had started, were allocated randomly to one of three groups. A control group was confronted with factual information. Meanwhile, those in the experimental groups saw the same information, but this time it was either supplemented with explicit references to its institutional sources or it contained these references and also used patronizing language. The content of the basic information was the same in each condition, as were subsequent questions in the survey.

### 3.3 Intervention design

The starting point for designing the experimental conditions was information taken from various pages on the website of Voedingscentrum, an independent organization communicating nutrition information on a national and regional level. This was supplemented with information obtained from other members of the EPHNA and Dutch health institutions like the Alliantie Voeding voor de Gezonde Generatie. The facts in the control condition were based on information on healthy drinks taken from the Voedingscentrum website, reduced to several core facts. The two experimental conditions presented the same information, but included either: 1) explicit references to its institutional sources; or 2) referred to these sources and used language that may be perceived as patronizing.

For these, we consulted the web pages of EPHNA members that explained how various food guidelines are produced, in particular relating to issues like funding and the acquisition of source material. We centered pedantic and imperative language in the second experimental condition, as well as the suggestion that the proposed behavior is the ‘proper’ approach. This was based on the various uses of language employed in the campaigns of the referenced institutions.

The use of examples from real-life nutrition information ensures the external validity of the study. However, as a single exposure to an information treatment in an experimental setting cannot compare to its continuous use in real-world scenarios, we adopted the common strategy of increasing the overtness of the manipulations (see Gaines et al., 2007). The institutional and patronizing elements in the two treatments were therefore slightly exaggerated.

### 3.4 Measures

Unless otherwise noted, all the questions were answered on a seven-point scale from (1) completely disagree to (7) completely agree.

*Perceived threat to freedom* was measured with four items: 1) My freedom to choose is taken away; 2) a choice is made for me; 3) something is forced upon me; 4) I am pressured into something. Spear-headed by the research of Dillard and Shen (2005), this measure is often used in studies of reactance, albeit with some alterations in the wording. An internally consistent scale (Cronbach's alpha = 0.90) was constructed using the mean score of the respondents who provided valid answers to all four questions. Higher scores on the scale indicated a greater perceived threat to freedom.

We also followed Dillard and Shen's (2005) research for *state reactance*, viewing it as a latent construct underlying both negative emotions and negative cognition. This dual measure is the best way to capture state reactance, as subsequently validated by Quick and Stephenson (2007). We therefore asked our respondents to indicate their level of anger, annoyance, irritation, and aggravation on a seven-point response scale, ranging from: (1) a great deal of this feeling to (7) none of this feeling. After reverse coding, an internally consistent scale (Cronbach's alpha = 0.96) was constructed using the mean score, with higher scores indicating a stronger emotional reactance. We adopted Al-Ghaithi et al.’s (2019) approach for the negative cognitions, which were measured with a Likert scale. In addition to the practical advantages of such a scale over the original thought-listing exercise, recent comparative research has confirmed a minor advantage of this approach (Reynolds-Tylus et al., 2021). The respondents were asked to evaluate their thoughts while reading the information using three seven-point Likert scale items: (1) unpleasant to (7) pleasant; (1) unfavorable to (7) favorable; and (1) negative to (7) positive. This produced an internally consistent scale (Cronbach's alpha = 0.94), which was constructed by reverse coding and taking the mean score for these items. Higher scores indicated a stronger cognitive reactance.

Various studies have determined that an ‘Intertwined Process Model’ is the best way to combine the two (Dillard & Shen, 2005; Quick & Stephenson, 2007; Rains & Turner, 2007), leading to a model in which they function as “indicators of an underlying concept” (Dillard & Shen, 2005, p. 149) – i.e., state reactance. Consequently, the measures for emotion and cognition were combined in a single scale. We tested internal consistency for the state reactance scale based on the standardized coefficient alpha. This is viewed as the most appropriate test for two-item scales (Eisinga et al., 2013) and indicated that our scale was internally consistent (Cronbach's alpha = 0.66). The final variable was calculated by taking the mean scores of emotional and cognitive reactance scales, with higher scores indicating that the state reactance was stronger.

The respondents' *negative attitude toward drinking sugar-sweetened beverages* was measured with three items, again based on a common measure used in reactance studies (cf., Dillard & Shen, 2005). To uncover whether the information caused the respondents to become more recalcitrant, and so more positive about the behavior it cautioned against, we asked them to indicate if they thought the idea of reducing their SSB intake (i.e., the core message) was (1) very bad to (7) very good; (1) very unnecessary to (7) very necessary; and (1) very unwise to (7) very wise. We reverse coded the items and created a single scale that was internally consistent (Cronbach's alpha = 0.87), using the mean score of the respondents who provided valid answers to each question. Higher scores indicated a more negative attitude.

*Intended non-compliance* is a single item measuring if the respondents planned to reduce their consumption of SSBs after reading the information. An additional answer category, coded as missing, was included for them to indicate whether they were already non-consumers: (8) I do not drink any sugar-sweetened beverages. The variable was reverse coded, meaning that higher scores indicated a higher level of intended non-compliance.

We used five items to measure the extent of the respondents' lack of identification with those they perceived to be the source of the information. Three items were derived from Chang (2011), while (the latter) two were newly added to expand the scale by encompassing a sociocultural element. These five items were: ‘The people from whom the information originates … ’ 1) are similar to me; 2) and I are alike; 3) and I could be friends; 4) share my norms and values; and 5) have the same outlook on life as me. The variable for *source disidentification* was created by reverse coding the items and creating a scale (Cronbach's alpha = 0.91) using the average score of the respondents who provided valid answers to each of the five questions. Higher scores indicated more disidentification.

*Source disappreciation* was adapted from McCroskey and Teven (1999). To avoid repetition in the Dutch translations of the items, the original 18 were reduced to nine. These were as follows: “The people from whom the information originates … 1) are smart; 2) know a lot about the subject; 3) are experts; 4) care for me; 5) want what is best for me; 6) are trying to help me; 7) are honest; 8) are trustworthy; 9) are sincere. A single, reverse coded, internally consistent scale (Cronbach's alpha = 0.93) was calculated for the respondents who provided valid responses to all nine items. A higher score on the scale indicated a stronger disappreciation of the perceived source.

Lastly, we measured the respondents' *negative attitude toward information provision* using three items. We asked the respondents to indicate if they thought providing information as a way to reduce the consumption of SSBs was (1) very bad to (7) very good; (1) very unnecessary to (7) very necessary; and (1) very unwise to (7) very wise. We reverse coded the items and created a single scale (Cronbach's alpha = 0.92), using the mean score of the respondents who provided valid answers to each question. Higher scores indicated a more negative attitude.

Our independent variables measured which treatment had been assigned to a respondent: (0) control condition; (1) information stressing institutional sources; (2) information stressing informational sources and using patronizing language. This was included in the analyses as dummy variables, with (0) as the reference category.

To test hypothesis 8, we interacted the treatment variables with our measure of educational attainment. The original levels were (1) primary school; (2) vmbo (intermediate secondary education); (3) havo/vwo (higher secondary education/preparatory university education); (4) mbo (intermediate vocational education, US: junior college); (5) hbo (higher vocational education); (6) wo (university); (7) other; (8) not (yet) completed any education; and (9) not yet started any education. Respondents that had a degree but were currently still in education (*n* = 92) were excluded from the analyses, as were respondents that answered, (7), (8) and (9) of the original measurement (*n* = 53). As recent studies have shown that there is a difference in the attitudes toward institutions between (mainly) those with a tertiary education on the one hand, and those with a non-tertiary education on the other (Van Meurs et al., 2022b; Noordzij et al., 2021b), we recoded this variable into two categories: (0) more educated (categories 5 and 6 of the original measurement); and (1) less educated (categories 1 to 4 of the original). We chose ‘more educated’ as the reference category for reasons of clarity, as our theoretical focus in the moderation hypotheses was on less-educated individuals.

### 3.5 Analytical strategy

We used ordinary least squares (OLS) regression models to identify the effects on our outcome measures of stressing institutional connections and the use of patronizing language.

For our main confirmatory analyses, we fitted a separate, but similar, model for each outcome measure:

(1) *Y = β*_*0*_*+ β*_*1*_*institutional + β*_*2*_*institutionalpatronizing +* ε

where Y is the outcome measure; *institutional* is a dichotomous variable indicating the treatment in which institutional sources are stressed; *institutionalpatronizing* is a dichotomous variable for the treatment that highlights institutional sources and also uses patronizing language; and ε is the error term. The treatment effects were compared to the control condition, which was the reference category. The equation was used to test both the ‘a’ and ‘b’ versions of the main effect hypotheses.

We also fitted a separate model for each outcome measure for our confirmatory moderation analyses:

(2) *Y = β*_*0*_*+ β*_*1*_*institutional + β*_*2*_*institutionalpatronizing + β*_*3*_*lessedu* + *β*_*4*_(lessedu*institutional) + β_*5*_(lessedu*institutionalpatronizing) + ε

where Y is the outcome measure; *institutional* is a dichotomous variable indicating the treatment variant in which institutional sources are stressed; *institutionalpatronizing* is a dichotomous variable indicating the treatment variant in which institutional sources are stressed and patronizing language is used; *lessedu* is a dichotomous variable indicating whether a respondent is less educated (1) or more educated (0); (lessedu*institutional) is the interaction between educational attainment and the first treatment; (lessedu*institutionalpatronizing) is the interaction between educational attainment and the second treatment; and ε is the error term. The treatment effects were compared to the control condition, which was the reference category. The equation above was used to test both the ‘a’ and ‘b’ versions of the moderation hypotheses.

## 4 Results

Our sample counted 1947 respondents after exclusions based on the time spent on the page of text, and educational attainment. The median age was 59 (mean age 57), 54 percent was female, and 56 percent was non-tertiary educated. The descriptive statistics for all the variables included in our analysis are reported in the Online Appendix (A1).

Overall, the experimental conditions had little effect on receptivity to the information provided (see Table 1). Perceived threat to freedom was the only outcome variable affected in any significant way by one of the experimental conditions in the direction hypothesized. In particular, the combination of stressing institutional sources and using patronizing language (compared to the control condition) caused a 0.14 increase in the threat perceived, corroborating hypothesis 1b. However, the same outcome variable was not affected by information that only stressed institutional sources, meaning hypothesis 1a was not corroborated. Hypotheses 2 to 7 were also not corroborated. Although both experimental conditions (compared to the control) did have a significant effect on source disappreciation, this was in the opposite direction to that hypothesized, causing a greater appreciation of the perceived informational sources.

**Table 1:** OLS regression for hypotheses 1 to 7; unstandardized coefficients with standard errors in parentheses.

	Reactance				Source derogation		
	H1: Perceived threat to freedom	H2: State reactance	H3: Negative attitude towards decreasing SSB-consumption	H4: Intended non-compliance	H5: Source disidentification	H6: Source disappreciation	H7: Negative attitude toward information provision
*Control condition*	Ref.	Ref.	Ref.	Ref.	Ref.	Ref.	Ref.
*Institutional condition*	−0.02 (0.07)	0.02 (0.06)	0.07 (0.06)	0.00 (0.13)	0.03 (0.06)	−0.19*** (0.05)	0.02 (0.06)
*Institutional and patronizing condition*	0.14* (0.07)	0.09 (0.06)	0.05 (0.07)	−0.09 (0.13)	0.02 (0.07)	−0.19*** (0.06)	0.01 (0.06)

Constant	2.26*** (0.05)	2.45*** (0.04)	1.98*** (0.05)	3.55*** (0.09)	3.53*** (0.05)	3.26*** (0.04)	1.97*** (0.04)

***p < 0.001, **p < 0.01 *p < 0.05.

Note: *n* = 1947 for all models except for intended non-compliance (H4; *n* = 1138). The lower *n* is due to the number of respondents that indicated “I do not drink any sugar-sweetened beverages” when asked about their intention to decrease their SSB consumption.

**Fig. 1:**
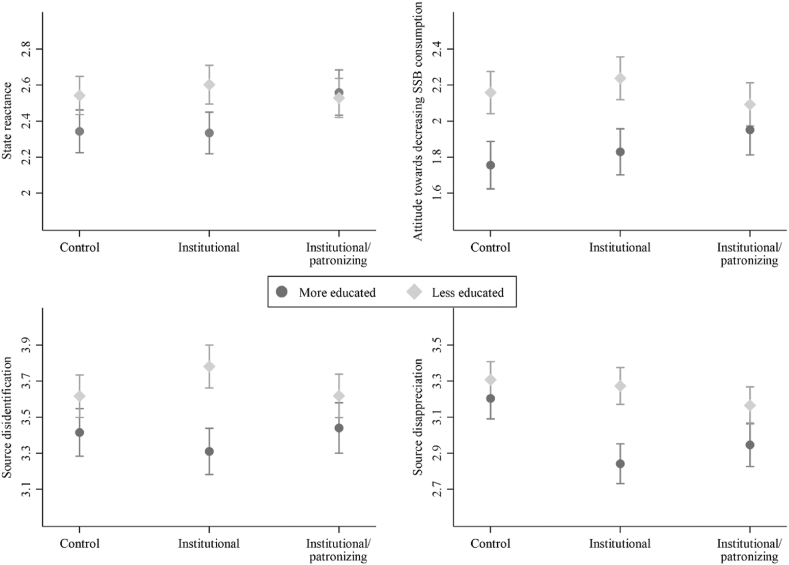
Visualization of significant moderation effects.

The moderation analysis in Table 2 shows that there were heterogeneous treatment effects (HTEs) for some of the outcome variables: four of the 14 interaction effects yielded significant coefficients. We plotted these in Fig. 1, which reveals that, contrary to our hypothesis, it was the more-educated citizens in particular whose receptivity was affected by the stimuli.

**Table 2:** OLS regression for hypothesis 8; unstandardized coefficients with standard errors in parentheses.

	Reactance				Source derogation		
	H8i: Perceived threat to freedom	H8ii: State reactance	H8iii: Negative attitude towards decreasing SSB-consumption	H8iv: Intended non-compliance	H8v: Source disidentification	H8vi: Source disappreciation	H8vii: Negative attitude toward information provision
Experimental condition							
*Control condition*	Ref.	Ref.	Ref.	Ref.	Ref.	Ref.	Ref.
*Institutional condition*	−0.07 (0.10)	−0.01 (0.08)	0.08 (0.09)	−0.04 (0.20)	−0.11 (0.09)	−0.36*** (0.08)	−0.00 (0.09)
*Institutional and patronizing condition*	0.19 (0.11)	0.22* (0.09)	0.20* (0.10)	0.02 (0.20)	0.02 (0.10)	−0.26** (0.08)	0.09 (0.10)

Less educated	0.46*** (0.10)	0.20* (0.08)	0.40*** (0.09)	−0.06 (0.18)	0.20* (0.09)	0.10 (0.08)	0.33*** (0.09)

Institutional * Less educated	0.12 (0.14)	0.07 (0.11)	0.01 (0.13)	0.07 (0.26)	0.27* (0.13)	0.33** (0.11)	0.06 (0.12)
Institutional and patronizing * Less educated	−0.10 (0.14)	−0.23* (0.12)	−0.26* (0.13)	−0.19 (0.26)	−0.02 (0.13)	0.12 (0.11)	−0.16 (0.13)

Constant	2.00*** (0.07)	2.34*** (0.06)	1.76*** (0.07)	3.59*** (0.14)	3.41*** (0.07)	3.20*** (0.06)	1.78*** (0.07)

***p < 0.001, **p < 0.01 *p < 0.05.

Note: *n* = 1947 for all models except for intended non-compliance (H4; *n* = 1138). The lower *n* is due to the number of respondents that indicated “I do not drink any sugar-sweetened beverages” when asked about their intention to decrease their SSB consumption.

Among the more-educated respondents, information that emphasized institutional sources and used patronizing language increased their levels of state reactance and negative attitudes toward reducing the consumption of SSBs (see Fig. 1); this was not the case for their less-educated counterparts. In terms of source derogation, the HTE was in line with hypothesis 8v-a: less-educated respondents confronted with information that stressed its institutional sources reported higher levels of source disidentification than those in the control condition, while their more-educated counterparts did not. However, this was not the case when they were confronted with information that also used patronizing language. Lastly, the stimuli did not lead to more source disappreciation among the less-educated citizens (as had been hypothesized); instead, it actually led to less source disappreciation among the more-educated respondents.

The outcomes of the moderation analysis mean that 13 of the 14 hypotheses regarding the strength of the information and source effects among the less-educated respondents must be rejected. Nonetheless, Fig. 1 also reveals a pattern worth further exploration: in all four depicted cases, the less-educated respondents were less receptive to nutrition information. As an additional explorative investigation, we conducted regression analyses with education as the predictor (see Table S2 in the Online Appendix). Save for intended non-compliance, less-educated respondents had more strongly negative scores for all the outcome measures than their more-educated counterparts. However, the testing of our hypotheses identified that this was not aggravated systematically by confronting them with information that also stressed its institutional sources or used patronizing language.

## 5 Discussion

The goal of this study was to uncover how receptivity to nutrition information was affected by two dominant communication strategies: emphasizing institutional sources, and simplifying information, with the unintended consequence of making it sound patronizing. While there have been plenty of warnings that people become more closed off to persuasive communication (cf., Rains, 2013), our study provides little to no evidence that this really occurs among the population at large, at least for the strategies tested in this study. Our use of population-based data from the Netherlands in this preregistered experiment has demonstrated that there is only a small negative effect on receptivity if information is presented in which institutional sources are emphasized *and* language is used that is generally perceived to be patronizing: only one of seven outcome measures – perceived threat to freedom – was slightly affected. Indeed, information that only stressed institutional sources to make it more persuasive did not affect receptivity negatively at all.

Studies in the field of psychological reactance have been a critical voice on the use of persuasive information, arguing that it could produce negative reactions, with boomerang effects being the most extreme outcome (Brehm & Brehm, 1981). Our results run counter to this, which may be due to sample differences – reactance studies largely use student-based convenience samples, often involving those enrolled in communication courses (e.g., Dillard & Shen, 2005). This closer connection to the subject-matter may produce biased, more critical views on the issue. Nonetheless, our experiment has shown that the postulated negative effect of persuasive communication on receptivity to nutrition information among the public is negligible.

In terms of the expectation that a negative effect is more likely among less-educated citizens, this was also barely in evidence: only one of the two experimental conditions (stressing institutional sources) had a stronger negative effect on only one measure of receptivity (source disidentification) among the less-educated respondents compared to their more-educated counterparts.

Two further observations should be made regarding the differences between less- and more-educated citizens in our experiment. First, our exploratory analysis demonstrated that the less-educated individuals in our sample were, overall, less receptive to health advice: they had higher scores for most outcomes, largely regardless of the experimental condition to which they were assigned. This is in line with previous studies that have shown that less-educated individuals are less interested in using health information (Van Meurs et al., 2022b; Koç & van Kippersluis, 2017).

Second, contrary to our expectations, receptivity to nutrition information containing patronizing language had a more negative impact on the more-educated respondents for two of our outcome measures (state reactance and negative attitudes toward reducing SSB consumption). This may be because this group perceives the language to be more condescending than is the case for their less-educated counterparts. Indeed, the former often have a greater appreciation of individual liberties and self-actualization (Beck & Beck-Gernsheim, 2002; Houtman et al., 2011, 2021) and may therefore detest being told what to do – especially if this is done in a manner that is so simplified and direct that it implies they lack the relevant knowledge. Moreover, indications in previous studies that less-educated individuals especially feel patronized by (health) professionals and institutions (Bergman et al., 2020; Noordzij et al., 2021b) might be due to very different factors than the type of language. Future research could shed light on this.

This study has some important implications for conventional persuasive-information strategies. Theoretical and empirical evidence, specifically that from the field of psychological reactance and source derogation, paints persuasive communication and the strategies involved as potentially endangering the intended effects of health-promotion efforts, as it may in fact lead to stronger aversion, rather than stronger compliance (cf., Rains, 2013). However, our findings show that conventional health-communication strategies have no negative effects overall on receptivity to the information being presented. Nonetheless, in this also lies a limitation of our study: due to the setup of our survey experiment, we were able to test for effects on attitudes but not on behavior. Consequently, future research should shift its focus toward behavioral – rather than attitudinal – change to identify the overall merit of the strategies discussed in this study.

A second limitation relates to the subject-matter of the information read by the respondents. As the responses to the non-compliance question show, a substantial proportion of them indicated that they were not consumers of SSBs. This may have affected receptivity in at least two ways: respondents 1) regard information about SSBs as personally irrelevant and are, therefore, not as affected by changes in communication strategies as they might otherwise be; or 2) are already against the high consumption of SSBs (see the low average score for negative attitudes toward both reducing SSB consumption and information to facilitate it). Therefore, they adopt the view that something should be done about this, making them likely to agree more with advice voiced authoritatively. Future research could thus investigate whether and how receptivity changes if the conventional strategies tested are applied to information about more ‘controversial’ topics, e.g., meat consumption. In addition, this study could be replicated in countries with a higher SSB consumption, like the United States or various Central American countries (Singh et al., 2015).

Results may also differ in countries where institutions are considered less legitimate in general. Perceived legitimacy of institutions is relatively high in the Netherlands, especially compared to e.g., countries in eastern and central Europe (Boda & Medve-Bálint, 2014). In such countries, persuasive communication by institutions may be more likely to create aversion. An international comparison might shed light on this.

A final limitation is the external validity of our survey experiment, as respondents may simultaneously be under- and overexposed to the treatment condition in comparison to its real-world comparison, and our setup does not allow us to ascertain which is the case. On the one hand, a one-shot treatment cannot capture accumulated effect of continuous exposure to real-world health information campaigns (Gaines et al., 2007). On the other hand, a survey experiment enhances the potency of the exposure by forcing respondents to pay more specific attention to the information, whereas real-world exposure is likely to be more fleeting (Barabas & Jerit, 2010). Future research may take exposure levels into consideration, study other contexts, or use other study designs for triangulation, in order to assess the external validity of the results.

## 6 Conclusion

In short, it can be concluded that emphasizing institutional sources in nutrition information and using simplified language that could be perceived as patronizing seem to be safe health-communication strategies. While they do not generally increase the receptivity to health information, neither do they substantially antagonize the recipients in any substantial way, contrary to the claims often made in reactance studies. It is crucial for future research to confirm and expand on our findings in order to identify the effects on actual behavioral change of stressing institutional sources and using patronizing language.

## Declarations of interest

None.

## Financial disclosure

The study was funded by the Erasmus Initiative ‘Smarter Choices for Better Health’. They had no role in the conceptualization of the study.

## Ethical statement

All subjects gave their informed consent for inclusion before they participated in the study. The protocol was accepted by the ethical committee of the Erasmus University Rotterdam.

## CRediT statement

**Tim van Meurs**: Conceptualization, Formal analysis, Writing – Original Draft **Joost Oude Groeniger**: Conceptualization, Formal analysis, Writing – Review & Editing, Funding acquisition **Willem de Koster**: Conceptualization, Writing – Review & Editing, Funding acquisition **Jeroen van der Waal**: Conceptualization, Writing – Review & Editing, Funding acquisition.

## Data Availability

Data will be made available on request.

## Footnotes

1 See http://www.ephna.eu/. EPHNA consists of 17 members from Germany, Belgium, Austria, Switzerland, Portugal, Spain, Denmark, Bulgaria, Poland, Malta, Greece, Latvia, Ireland, the Netherlands, Czech Republic and Italy.

2 See https://www.Voedingscentrum.nl/nl.aspx.

3 Please note that while the hypotheses in the manuscript correspond to those in the preregistration (except for some stylistic adjustments), their order (and therefore their numbers) differ.
