# Supplementary material for: Receptive to an authoritative voice? Experimental evidence on how patronizing language and stressing institutional sources affect public receptivity to nutrition information
Source: SSM Popul Health. 2022 Nov 19;20:101295. doi: 10.1016/j.ssmph.2022.101295 (PMC9706606; doi:10.1016/j.ssmph.2022.101295)
Supplement: Multimedia component 1 [file mmc1.docx]

**Online Appendix**

**A1 – Descriptive statistics**

| **Table S1 –** Descriptive statistics | | | | | |
| --- | --- | --- | --- | --- | --- |
|  | *n* | Mean | S.D. | Min | Max |
| Perceived threat to freedom | 1,947 | 2.30 | 1.26 | 1 | 7 |
| State reactance | 1,947 | 2.49 | 1.05 | 1 | 7 |
| Neg. attitude towards decreasing SSB consumption | 1,947 | 2.02 | 1.17 | 1 | 7 |
| Intended non-compliance | 1,138 | 3.52 | 1.78 | 1 | 7 |
|  |  |  |  |  |  |
| Source disidentification | 1,947 | 3.54 | 1.17 | 1 | 7 |
| Source disappreciation | 1,946 | 3.14 | 1.01 | 1 | 7 |
| Neg. attitude towards information provision | 1,947 | 1.98 | 1.15 | 1 | 7 |
| Experimental condition |  |  |  |  |  |
| *Control condition* | 1,947 | 0.34 |  | 0 | 1 |
| *Institutional condition* | 1,947 | 0.34 |  | 0 | 1 |
| *Institutional and patronizing condition* | 1,947 | 0.32 |  | 0 | 1 |
| Education attainment |  |  |  |  |  |
| *Less educated* | 1,947 | 0.56 |  | 0 | 1 |
| *More educated* | 1,947 | 0.44 |  | 0 | 1 |

**A2 – Explorative analysis**

**Table S2** – Ordinary Least Squares regression analysis for education differences at baseline, unstandardized coefficients

|  | Reactance | | | | Source derogation | | |
| --- | --- | --- | --- | --- | --- | --- | --- |
|  | Perceived threat to freedom | State reactance | Negative attitude towards decreasing SSB-consumption | Intended non-compliance | Source disidentification | Source disappreciation | Negative attitude towards information provision |
| More educated | Ref. | Ref. | Ref. | Ref. | Ref. | Ref. | Ref. |
| Less educated | 0.48***  (0.06) | 0.15***  (0.05) | 0.32***  (0.05) | -0.10  (0.11) | 0.29***  (0.05) | 0.25***  (0.05) | 0.30***  (0.05) |
|  |  |  |  |  |  |  |  |
| Constant | 2.03***  (0.04) | 2.40***  (0.04) | 1.84***  (0.04) | 3.58***  (0.08) | 3.38***  (0.04) | 3.00***  (0.03) | 1.81***  (0.04) |

***p < 0.001, **p < 0.01 *p < 0.05

Note: *n* = 1,947 for all models but intended non-compliance (H4; *n* = 1,138). The lower *n* in this model is due to the high number of respondents that indicated “I do not drink any sugar-sweetened beverages” when asked about their intention to decrease their SSB consumption.
